# Supplementary material for: Observation of dissociative quasi-free electron attachment to nucleoside via excited anion radical in solution
Source: Nat Commun. 2019 Jan 9;10:102. doi: 10.1038/s41467-018-08005-z (PMC6327028; doi:10.1038/s41467-018-08005-z)
Supplement: Supplementary file 1 — Supplementary Information [file 41467_2018_8005_MOESM1_ESM.pdf]

Supplementary Information (SI)

Observation of dissociative quasi-free electron attachment to nucleoside via excited anion radical in solution

Jun Ma[1*], Anil Kumar[2], Yusa Muroya[3], Shinichi Yamashita[4], Tsuneaki Sakurai[1], Sergey A. Denisov[5], Michael D. Sevilla[2], Amitava Adhikary[2], Shu Seki[1*], Mehran Mostafavi[5*]

[1] - Department of Molecular Engineering, Graduate School of Engineering, Kyoto University, Nishikyo-ku, Kyoto 615-8510, Japan

[2]- Department of Chemistry, 146 Library Drive, Oakland University, Rochester, Michigan 48309, USA

[3] - Department of Beam Materials Science, Institute of Scientific and Industrial Research, Osaka University, 8-1 Mihogaoka, Ibaraki, Osaka 567-0047, Japan

[4] - Nuclear Professional School, School of Engineering, The University of Tokyo, 2-22 Shirakata Shirane, Tokai-mura, Naka-gun, Ibaraki 319-1188, Japan

[5]- Laboratoire de Chimie Physique, UMR 8000 CNRS/Université Paris-Sud, Bât. 349, Orsay 91405 Cedex, France

1.1.Supplementary Note 1 Picosecond pulse radiolysis setups

For the present study, broadband probe transient absorption pulse-probe setups were used: Nuclear Professional School of the University of Tokyo (18 MeV, 10-20 Hz, 15 FWHM, 2 nC) (1, 2) and ELYSE (Univeristy of Paris-Sud, France; 8MeV, 10-20Hz, 5 ps FWHM, 4 nC). A supercontinuum, generated by focusing ~1 µJ of the laser source (100 fs) into a 6-mm-thick CaF_2 disk, was used as the optical probe covering a broad spectral range (France: 370 to 800 nm and Japan: 370-1050). A reference signal was split off from the broadband probe before the fused silica optical flow cell. Japan: The probe light was detected by a multi-channel analyzer (PMA20, Hamamatsu Photonics Co. Ltd.) which consists of 2048 channels of CCD and covers the wavelength of 350-1100 nm. The broad absorption band of hydrated electron was well-established, and it was used as a "reference" to check the reliability of our results from the supercontinuum. As shown in Fig. SI1, the spectrum of hydrated electron from 370 to 1050 nm measured in water is identical with the reported one (3). France: Each of the probe and reference beams was coupled into the optical fibers, transmitted to a spectrometer, and dispersed onto a charge-coupled device (Andor Newton 920). The combination of the broadband probe and the multichannel detector allowed the entire transient spectrum to be recorded in one shot; as a result, the transient spectrum was independent of the shot-to-shot fluctuations and possible long-term drifts of the electron source. For data acquisition, we cautiously maintained the same radiation dose (55.3 ± 0.5 Gy) per electron pulse that was deposited on the samples to minimize the absorbance fluctuation. All measurements were performed using a fused silica flow cell with a 0.5 or 1 cm optical path collinear to the electron pulse propagation at 22.5°C.

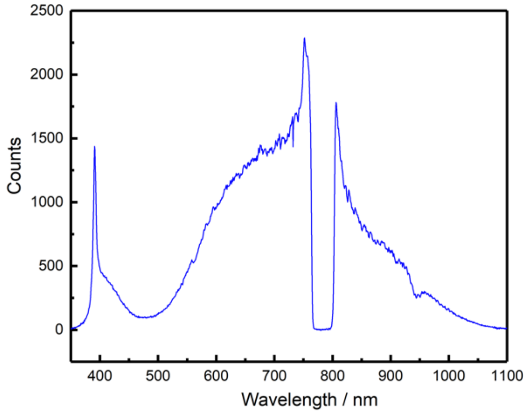

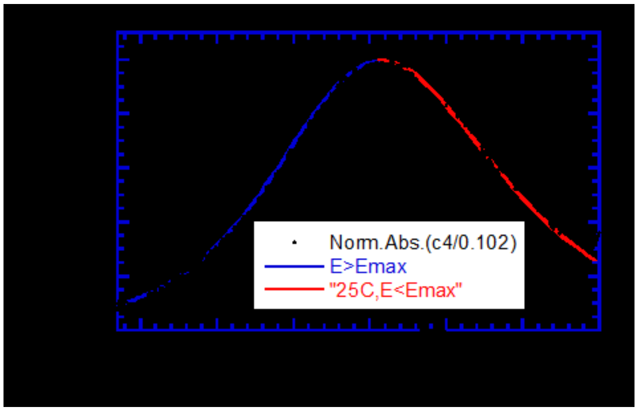

Supplementary Figure 1: The supercontinuum generated by CaF_2 at Japan LINAC facility (Left). The transient absorption spectrum of hydrated electron (e_{sol}^-) ranging from 370 to 1050 nm (scattering black points) in comparison with the one in literature (right), showing the reliability of the absorption data from the supercontinuum. e-beam (D = 4mm) and probe light are perpendicularly crossed at the sample cell (1 cm × 1 cm) to avoid Cherenkov light and to reduce absorption of quartz window as much as possible.

1.2.Nanosecond pulse radiolysis setup

Nanosecond pulse radiolysis experiments were carried out at the S-band electron linear accelerator, the same facility at the University of Tokyo. Details of the apparatus for pulse radiolysis were described elsewhere (4, 5). The electron pulse has an energy of 35 MeV, and its width was 10 ns with a dose of 10–70 Gy per pulse. The dose per pulse at room temperature was determined using N_2O-saturated 0.01 M KSCN solution and $G\varepsilon([(SCN)_2^{-\bullet}])$ of 5.2×10^{-4} m^2 J^{-1} at 472 nm. The solutions subjected to radiation were constantly pumped with Ar inert gas to avoid the effects of oxygen. All the chemicals, 5-mehyluridine and diethylene glycol (purity> 99％) were purchased from Sigma Aldrich without further purification. All the measurements were performed at ambient condition.

2. Supplementary Note 2

2.1. Analysis of transient absorption profiles

We used a multivariate curve resolution alternating least squares (MCR-ALS) approach that aims at treating the 2D data, e.g. Fig. SI4 (6). This code allows us to assess the number of absorbing species in a data matrix and deconvolute their corresponding kinetics and spectra. It is often useful for the system containing three or more than three absorbing species. In our present work, a combination of both analysis approaches: global MCR-ALS analysis and simple subtraction (Fig. SI4) have been used to sort out the spectral characterizations of individual species. The analyzed results are consistent, and they lead to the same conclusion.

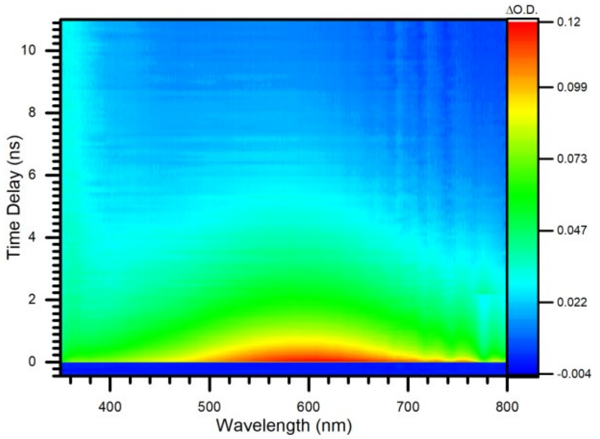

Supplementary Figure 2: A typical 2D experimental data of pulse radiolysis solution of 300 mM rT /DEG. The delay line in pump-probe is adjusted from 5 ps to 11 ns.

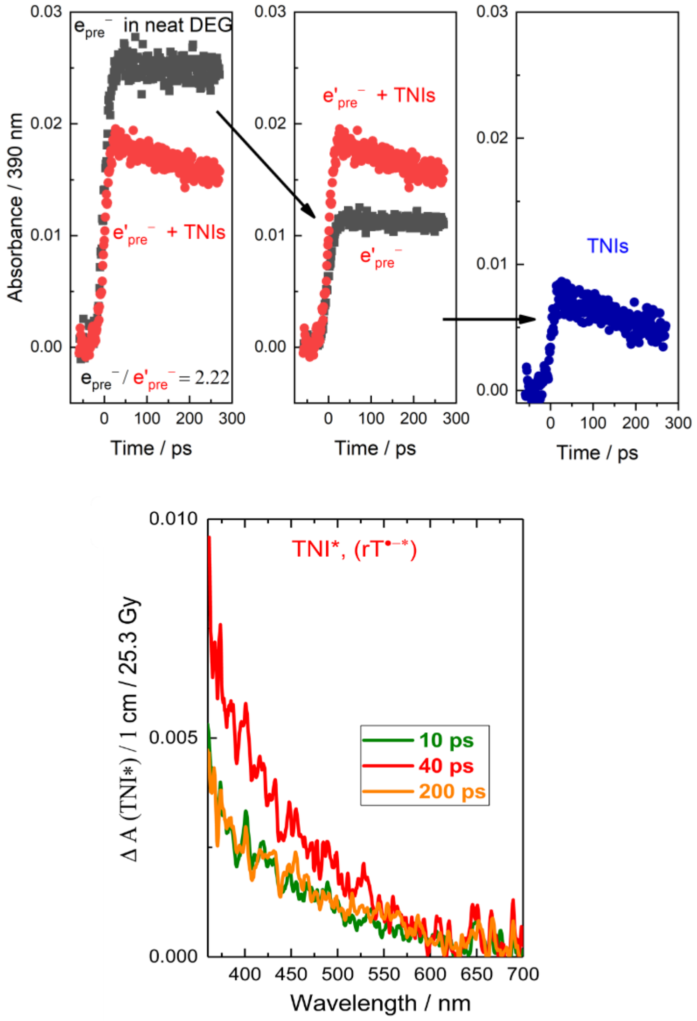

Supplementary Figure 3: Top: A schematic description of data treatment to obtain the kinetic of TNIs* in 0.3 M ribothymidine solution. In this solution, the transient absorption kinetics in the UV-visible region, for example, at 390 nm only includes the e_{pre}^- and TNIs absorption. Bottom: transient absorption spectra of TNIs* as a function of delay time.

2.2. Correlation between scavenged electrons with excited TNIs*

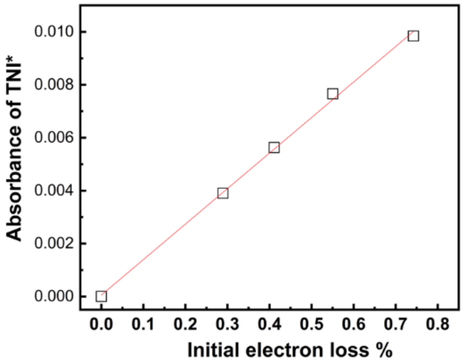

Supplementary Figure 4: The linear correlation of the percentage of initial electron loss captured by rT with the absorbance of TNI* in the UV region. The correlation supports our assignment that transient signal (see Figs. 1c and 3a) are the excited transient negative ions, TNIs* or ($rT^{\bullet-*}$), formed by e_{qf}^- attachment to rT.

2.3. Nanosecond pulse radiolysis

From our previous work (7), e_{pre}^- does not react with nucleotides in dilute solution below 50 mM. Thus, we carried out nanosecond pulse radiolysis to investigate the reaction of e_{sol}^- with ribothymidine to form ground state of TNIs, ($rT^{\bullet-}$). The transient absorption spectra difference as well as the lifetime between $rT^{\bullet-*}$ and $rT^{\bullet-}$ is obvious.

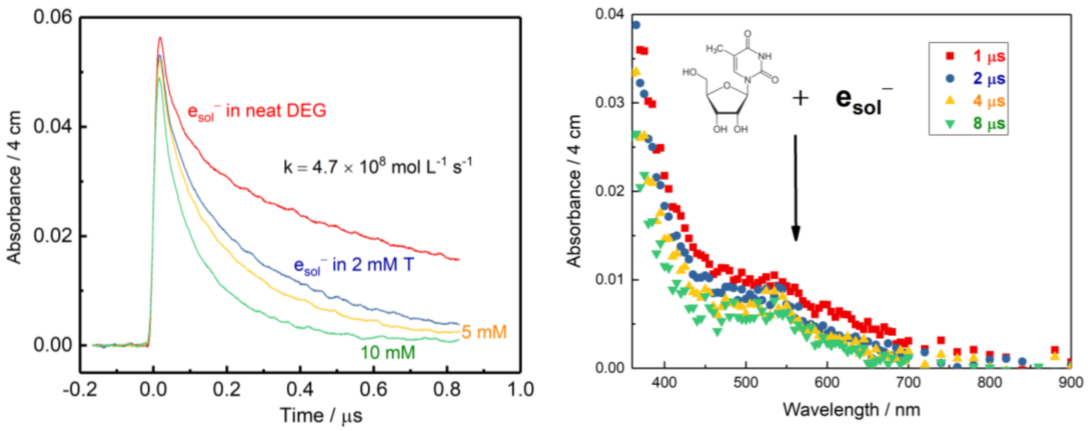

Supplementary Figure 5: Left: transient kinetics observed at 600 nm in dilute solutions of rT (2 to 10 mM) (left). The rate constant of reaction of e_{sol}^- with rT is ca. 4.7×10^8 $M^{-1}s^{-1}$. By considering the viscosity, the bimolecular reaction is shown to be controlled by diffusion. Right: transient absorption spectra of $rT^{\bullet-}$.

3. Supplementary Note 3 TD-DFT calculations of ribothymidine TNI excited states

3.1. Methods of calculation

It is well-known that a LEE on interacting with a molecule create transient negative ion (TNI) resonances which are equivalent to vertical excited states of the electron adduct of the parent molecule (8-11). A detailed knowledge about the formation of the TNI is of fundamental importance since reactions of LEEs such as dissociative electron attachment (DEA) lead to molecular fragmentation. The specific resonance energies available for direct capture of LEEs, can be found from the calculation of the transition energies to vertical excited states of a TNI. In the calculation of the excited states of a TNI, the proper choice of the basis set is very important since such resonances can be embedded in continuum. From our previous works (8-12), we note that the use of the compact basis set without diffuse functions is a good choice for such types of calculations (8-12). As a test, we earlier calculated the vertical transition energies of TNI of DNA/RNA bases in the gas phase using the time dependent (TD) density functional theory (DFT) and 6-31G* basis set (8, 9) and found that the calculated vertical transition energies match very well with shape resonance energies found experimentally by Aflatooni et al. (13) using electron transmission spectroscopy. In this present study, we used ωB97XD density functional and 6-31G** basis set and the effect of bulk solvent with dielectric constant of DEG ($\varepsilon = 31.69$) was incorporated via the use of integral equation formalism polarized continuum model (IEF-PCM).

The ground state geometry of ribothymidine in the neutral state was fully optimized using the ωB97XD density functional and 6-31G** basis set including the effect of bulk solvent with dielectric constant of DEG ($\varepsilon = 31.69$). For the calculation of the vertical excited state of TNI, we added an extra electron to the optimized neutral geometry of ribothymidine (no optimization of radical anion was done) and calculated the vertical excited states using the time-dependent (TD) variant of DFT (TD-DFT). Herein the complete methodology is abbreviated as TD-ωB97XD-PCM/6-31G**. The calculations were performed using the Gaussian 16 suite of programs (14).

In Fig. (SI6-SI9), the potential energy surface (PES) of N1-C1' bond dissociation of TNI of ribothymidine was scanned from its equilibrium bond length of neutral ribothymidine (vertical anion radical) to 2.1 Å in the step size of 0.1 Å using the ωB97XD-PCM/6-31G** method. At each constrained N1-C1' bond length on the PES the vertical excited states of TNI were also calculated using the TD-ωB97XD-PCM/6-31G** method. The vertical excited states of TNI of ribothymidine calculated in different environment (PCM and gas phase) are shown below.

3.2. Excited states of TNI of ribothymidine in the equilibrated PCM

Figures SI6 and SI7 show the vertical excited state as a function of the N1-C1′ bond distance in red. The fully optimized ground and excited states are shown in green. The dashed line shows the barrier free path from vertical excitation to the relaxed state with bond length increase.

The TD-ωB97XD-PCM/6-31G** calculated three lowest vertical transition energies of TNI are 1.3, 3.6 and 4.1 eV, respectively, and these transitions are $\pi \rightarrow \pi\sigma^*$, $\pi \rightarrow \sigma^*$ and $\pi \rightarrow \sigma^*$ types (see Fig SI10). Our TD-ωB97XD-PCM/6-31G** calculated first transition energy 1.3 eV is in excellent agreement (lowest resonance 1.2 eV localized on N1-C1' glycosidic bond) with those found experimentally by Illenberger and coworkers (15) for the decomposition of thymidine in the gas phase due to LEE, see Figure SI8. From the nature of the PES of the TNI ($^2\pi_{TNI}$, red curve), we see that as N1-C1' bond elongates the energy of ground state of TNI ($^2\pi_{TNI}$) surface increases until it crosses the

dissociative $^2\sigma^*$ surface with a barrier of 1.6 eV depicted in Fig. SI7. The energy of the first excited state $^2\pi \rightarrow ^2\pi\sigma^*$ ($^2\pi\sigma^*$ surface; red curve) also increases as N1-C1' bond elongates and at 1.8 Å it crosses the dissociative $^2\sigma^*$ surface having a barrier of ca. 1. eV. We note that dissociative $^2\sigma^*$ surface ($\pi \rightarrow \sigma^*$) initially lies at quite high energy (4.1 eV) but rapidly falls in energy on bond extension, see Figs SI6 and SI7. Thus, this surface is not initially accessible by e_{pre}^-, until the N1-C1' bond stretches and electron capture in the bond occurs and leads to dissociation. We, also, optimized the $^2\pi\sigma^*$ excited state (18) designated as $^2\pi\sigma^*_{OPT}$ in Fig. SI6 and SI7. From Fig. SI6, as expected $^2\pi\sigma^*_{OPT}$ lies lower than the vertical $^2\pi\sigma^*$ surface and after surpassing a small barrier ca. 0.6 eV, the N1-C1' bond dissociates. The blue dotted line in Fig. SI7, denotes a proposed barrier free dissociation path which occurs on capture of e_{pre}^- into the vertical πσ-MO of ribothymidine ($^2\pi\sigma^*$ surface) which on extension of the ~~C-N~~ N1-C1' bond relaxes from the vertical to the adiabatic surface.

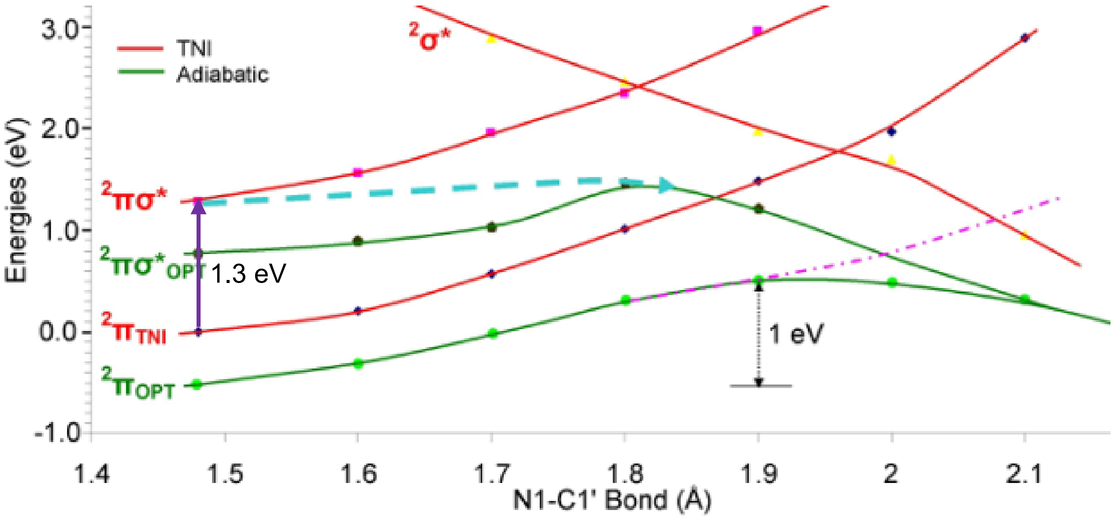

Supplementary Figure 6: Potential energy surface (PES) profiles for N1-C1' bond cleavage in ground and vertical excited states of ribothymidine transient negative ion (TNI). Red curves for the PES of TNI are designated as $^2\pi_{TNI}$; $^2\pi\sigma^*$ and $^2\sigma^*$. The X-axis shows the N1-C1' bond elongation. Green curves show the PES of adiabatic ground state ($^2\pi_{OPT}$) and optimized lowest excited state ($^2\pi\sigma^*_{OPT}$) of ribothymidine anion radical. The calculations are carried out using the TD-ωB97XD-PCM/6-31G** method including the equilibrated solvation with dielectric constant of DEG (ε = 31.69) via polarized continuum model (PCM). The dotted blue line is the proposed path leading to barrierless and fast N1-C1' bond dissociation by e_{qf}^- that begins in the excited state of TNI and moves on bond extension to the TNI relaxed barrier. Molecular orbitals of TNI involved in transition are shown in Fig. SI7. The calculated lowest vertical transition 1.3 eV from $^2\pi_{TNI} \rightarrow ^2\pi\sigma^*$ shown by purple arrow is in good agreement experiment value 1.2 eV, see reference 15.

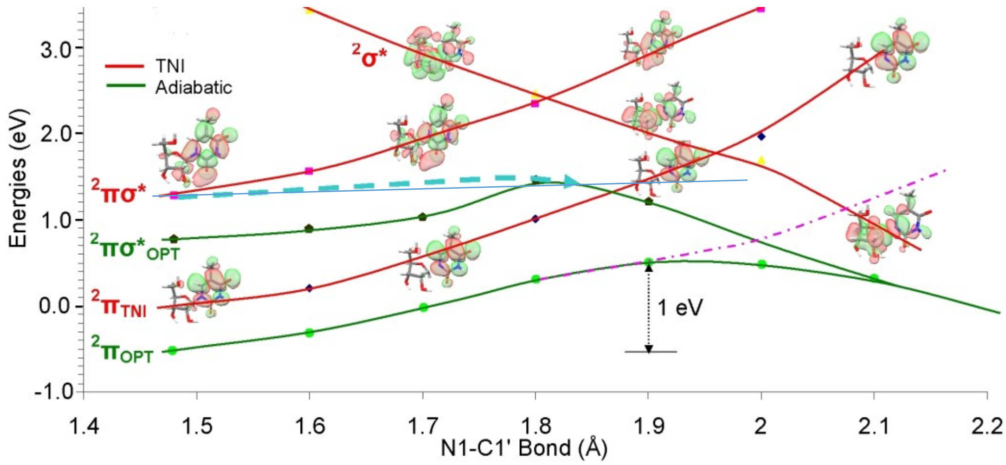

Supplementary Figure 7: Similar to Fig. SI6, the molecular orbitals involved in the transition from $^2\pi_{TNI}$ to $^2\pi\sigma^*$ and $^2\sigma^*$ are shown. For clarity MOs are not shown in Fig. SI6 above.

3.3. Excited states of TNI of ribothymidine in the non-equilibrated PCM

The excited states of the TNI considering the non-equilibrated solvent (using the PCM model) and we found that first excited state lies at 1.2 eV as in the gas phase. For calculation of the non-equilibrated solvent (PCM model), we optimized the neutral ribothymidine in PCM (equilibrated system) and saved the PCM coordinates and in TNI we used the saved equilibrated PCM coordinates and calculated the excites states.

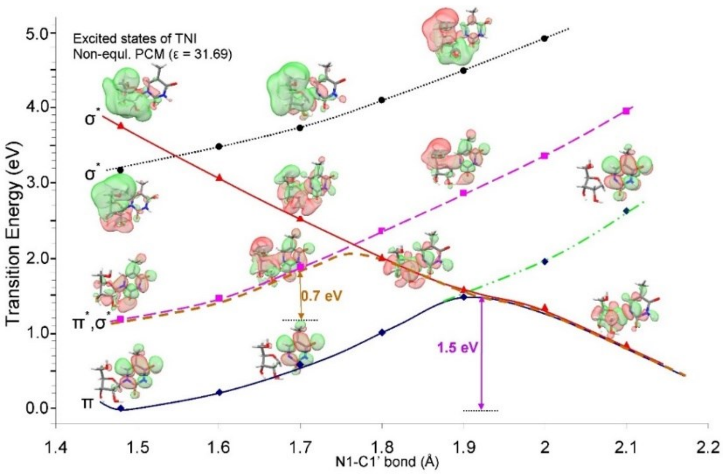

Supplementary Figure 8: Potential energy surface (PES) profiles for N1-C1' bond cleavage in ground and vertical excited states of ribothymidine transient negative ion (TNI) in the non-equilibrated PCM. The calculations are carried out using the TD-ωB97XD-PCM/6-31G** method.

3.4 Excited states of TNI of ribothymidine in the gas phase

Excited states of transient negative ion (TNI) of ribothymidine calculated in the gas phase using the TD-ωB97XD/6-31G** method. From Fig. SI9, it is evident that the first lowest excited state ($^2\pi^*$) couples with the dissociative $^2\sigma^*$ state very rapidly with increasing N1-C1' bond length and leads to immediate N1-C1' glycosidic bond dissociation. This state is about 1.2 eV above the ground state of the TNI.

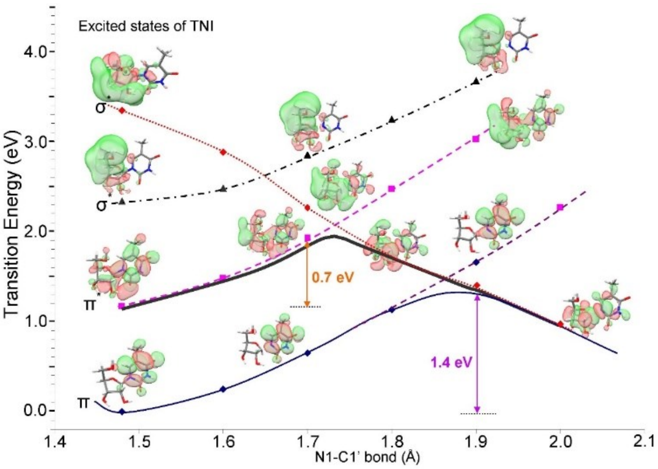

Supplementary Figure 9: Potential energy surface (PES) profiles for N1-C1' bond cleavage in ground and vertical excited states of ribothymidine transient negative ion (TNI) in the gas phase. The calculations are carried out using the TD-ωB97XD/6-31G** method. The black solid line shows the proposed path for N1-C1' glycosidic bond dissociation.

4. Supplementary References

1. Muroya, Y., Lin, M. Z., Iijima, H., Ueda, T. & Katsumura, Y. Current status of the ultra-fast pulse radiolysis system at NERL, the University of Tokyo. *Res. Chem. Intermed*. **31**, 261–272 (2005)
2. Muroya, Y.; Lin, M. Z.; Iijima, H.; Ueda, T.; Katsumura, Y. First observation of picosecond kinetics of hydrated electrons in supercritical water. *J. Phys. Chem. Lett*. **1**, 331–335 (2010)
3. D. M. Bartels.,Takahashi, K.,Jason A. Cline, J. A., Marin, T. W. & Jonah., C. D. Pulse radiolysis of supercritical water. 3. spectrum and thermodynamics of the hydrated electron. *J. Phys. Chem. A* **109**, 1299-1307 (2005).
4. Lin, M., Muroya, Y., Baldacchino, G. & Katsumura, Y. *Radiolysis of Supercritical Water, in Recent Trends in Radiation Chemistry*, ed. J. M. Wishart and B. S. M. Rao, (World Scientific, Singapore, ch. **9**, 231–253,2010)
5. Lin, M. &Katsumura, Y. Radiation Chemistry of High Temperature and Supercritical Water and Alcohols, in *Charged Particle and Photon Interactions with Matter – Recent Advances, Applications, and Interfaces*, ed. (Y. Hatano, Y. Katsumura and A. Mozumder, Taylor & Francis, Boca Raton, ch. 15, pp. 401–424 2010)
6. Ruckebusch, C., Sliwa, M., Pernot, P., De Juan, A. & Tauler, R. Comprehensive data analysis of femtosecond transient absorption spectra: A review. *J. Photochem. Photobiol. C* **13**, 1–27 (2012).
7. Ma, J., F. Wang, F., Denisov, S. A., Adhikary, A. & Mostafavi, M. Reactivity of prehydrated electrons toward nucleobases and nucleotides in aqueous solution. *Sci. Adv.* **3**. 1-7 (2017).
8. Kumar, A. &Sevilla, M. D. The role of πσ* Excited states in electron-induced DNA strand break formation: A Time-Dependent density functional theory study. *J. Am. Chem. Soc*. **130**, 2130-2131 (2008).
9. Kumar, A. & Sevilla, M. D. Role of excited states in low-energy electron (LEE) induced strand breaks in DNA model systems: Influence of aqueous environment. *Chem Phys Chem* **10**, 1426 – 1430 (2009).
10. Kumar, A. & Sevilla, M. D. Low-energy electron (LEE)-induced DNA damage: theoretical approaches to modeling experiment. In Handbook of Computational Chemistry, J. Leszczynski et al. (eds.), Springer Science+Business Media Dordrecht 2015, 1 – 63.
11. Schulz, G. J. Resonances in electron impact on diatomic molecules. *Rev. Mod. Phys*. **45**, 423–486 (1973).
12. Li, X., Cai, Z., & Sevilla, M. D. DFT Calculations of the electron affinities of nucleic acid bases: dealing with negative electron affinities. *J. Phys. Chem*. A **106**, 1596-1603 (2002).
13. Aflatooni, K., Gallup, G. A. & Burrow, P. D. Electron attachment energies of the DNA bases. *J. Phys. Chem. A* **102**, 6205- 6207 (1998).
14. Frisch, M. J et al. Gaussian 16; Gaussian Inc.: Wallingford, CT, 2009.
15. Ptasińska, S. et al. Decomposition of thymidine by low-energy electrons: Implications for the molecular mechanisms of single-strand breaks in DNA. *Angew Chem Int Ed*. **45**, 1893-1896 (2006).
